# A systematic review of trial-based economic evaluations of internet- and mobile-based interventions for substance use disorders

**DOI:** 10.1093/eurpub/ckz022

**Published:** 2019-07-12

**Authors:** Claudia Buntrock, Fanny Kählke, Filip Smit, David Daniel Ebert

**Affiliations:** 1 Department of Clinical Psychology and Psychotherapy, Friedrich-Alexander-University Erlangen-Nuremberg, Erlangen, Germany; 2 Trimbos Institute, Netherlands Institute of Mental Health and Addiction, Utrecht, The Netherlands; 3 Department of Clinical, Neuro and Developmental Psychology, Amsterdam Public Health Research Institute, VU University Medical Center, Amsterdam, The Netherlands; 4 Department of Epidemiology and Biostatistics, Amsterdam Public Health Research Institute, VU University Medical Center, Amsterdam, The Netherlands

## Abstract

**Background:**

Substance use disorders (SUDs) contribute significantly to global rates of morbidity and mortality. Internet- and mobile-based interventions (IMIs) have been suggested as an adjunct to face-to-face health services. However, the evidence for the cost-effectiveness of IMIs for SUDs is scant.

**Methods:**

A comprehensive literature search in PubMed, PsycINFO, the Cochrane Central Register of Controlled Trials, NHS Economic Evaluations Database, NHS Health Technology Assessment Database, Office of Health Economics Evaluations Database and EconLit was conducted. We included economic evaluations alongside randomized controlled trials of IMIs for SUDs compared with a control group.

**Results:**

Of 1687 abstracts identified, 11 studies met the inclusion criteria. Targeted conditions were alcohol use disorder (four studies) and tobacco smoking (five studies) whereas two studies included any SUD. Cost-effectiveness results demonstrated that IMIs had a firm probability of being more cost-effective than TAU (e.g. less costs per additional abstinent person). Compared with (online) psycho-education, evidence towards an additional benefit of IMIs was less clear. Regarding cost-utility (e.g. costs per quality-adjusted life year gained), except for one study, results suggested that TAU and online psycho-education would probably be more preferable than IMIs. Quality of study reporting was at least adequate.

**Conclusions:**

The likelihood of IMIs being more cost-effective than TAU looks promising but more economic evaluations are needed in order to determine the economic merit of IMIs. With an increasing pressure on health care budgets, strategies to disseminate effective interventions at affordable costs are required. This review suggests that IMIs might carry that promise and have potential as a cost-effective strategy to scale-up existing evidence-based treatments for SUDs.

**Systematic review registration:**

The systematic review has been registered in the PROSPERO database (no. CRD42018099486).

## Introduction

Substance use disorders (SUDs) including tobacco and alcohol contribute significantly to global rates of morbidity and mortality.^1^ Estimated 12-month prevalence of alcohol use disorder range from 11.8% in European regions^2^ to 12.7% in the US population.^3^ The hazardous and harmful use of alcohol is a global problem, contributing 4.6% of the total global burden of disease, with the highest rates reported in the European and American regions (17.3% and 14.2%, respectively).^4^ In 2014, overall prevalence rates of tobacco smoking were estimated at 27.2% in Europe.^5^ Tobacco smoking is a major preventable cause of death in both developed and developing countries.^6^ Smoking imposes a huge economic burden on society—currently up to 15% of the total healthcare costs in developed countries.^7^

There is robust evidence for the effectiveness of brief, face-to-face interventions in helping people to quit smoking^8^ and psychosocial treatments for substance abuse and dependence.^9^ In Europe, however, only 22.3% of alcohol dependent individuals seek professional treatment.^10^ Numerous impediments restrict the accessibility of available treatments, including costs, transport, inconvenience, fear of social- and work-related stigma and discrimination.^11^

Internet- and mobile-based interventions (IMIs) have been suggested to overcome many of these barriers to accessing traditional health services. In particular, IMIs can be anonymous and accessed whenever required, two factors that are especially relevant for SUDs.^12^ In addition, IMIs have demonstrated effectiveness for harmful alcohol use^13^ and smoking cessation.^6^^,^^8^ For harmful alcohol use, meta-analytic evidence showed a small but significant overall effect size in favour of IMIs compared with control conditions (g = 0.20, 95% CI: 0.13–0.27, *P* < .001).^13^ Recent meta-analytic evidence revealed an effect in favour of IMIs compared with non-active controls for smoking cessation (RR: 1.15, 95% CI: 1.01–1.30).^8^

Although the initial costs of designing, building and testing IMIs can be quite high, the low marginal costs of providing IMIs to additional users are believed to result in lower overall expenditures.^14^ In addition, IMIs are likely to reduce health service delivery costs compared with conventional face-to-face therapy, as they generally involve minimal or no contact with mental health professionals and reduce travel costs. IMIs are therefore assumed to be cost-effective, but it is unclear how strong this evidence is, and what the quality of this evidence is. This information is, however, critical for policymakers to allocate scarce health care resources.

Previous reviews on the economic evidence of IMIs, however, have focussed solely on physical illnesses,^15^ mood and anxiety disorders^16^^,^^17^ or IMIs based on cognitive behaviour therapy.^18^ The only systematic review on evidence-based IMIs for mental health problems and disorders (including harmful alcohol use and smoking cessation) consists of studies published up to 2014. However, more economic evaluations of IMIs for SUDs have been conducted in recent years. We therefore aimed to systematically review the available literature on trial-based economic evaluations of IMIs for SUDs in all age groups compared with control conditions.

## Methods

The current review was conducted in agreement with the Preferred Reporting Items for Systematic Reviews and Meta-Analyses (PRISMA).^19^ The protocol of this review is registered in the International Prospective Register of Systematic Reviews (PROSPERO; registration number CRD42018099486).

### Search strategy

Eligible cost-effectiveness studies were identified through a (PubMed) search in Medline, PsycINFO, Cochrane Central Register of Controlled Trials, NHS Economic Evaluations Database (NHSEED), NHS Health Technology Assessment (NHS HTA) and EconLit for articles published until 31 May 2018. Search terms indicative for SUDs, economic evaluations and IMIs were used (see Supplemental Data for full search string). References lists of previous systematic reviews and eligible studies were also examined to identify papers missed by database searches.

### Inclusion and exclusion criteria

Randomized controlled trials were included if they were comparative economic evaluations (e.g. cost-effectiveness and cost-utility analyses) of IMIs for the prevention or treatment of SUDs in all age groups published in English, German or Dutch. IMIs were defined as psychological interventions that were provided in an online setting, defined as internet-, online-, web- or mobile-based (with human support/guided or as a self-help intervention/unguided). Guidance usually consists of written non-therapeutic feedback by an e-Coach after a completed intervention module. The main purpose of the guidance is to encourage the participant to work through the self-help material independently. Studies were excluded if the IMI was offered as blended care, i.e. the combination of internet- and face-to-face treatment modalities.^20^ No exclusion criteria for comparator conditions were defined. Only full economic evaluations that reported comparisons of costs (including costs of interventions with or without costs beyond of the intervention) and outcomes of at least two alternatives were included. Cost-of-illness studies and descriptive economic studies only reporting costs without comparative outcomes or reporting costs and outcomes of only one intervention were excluded. Model-based economic evaluations were also excluded due to methodological differences compared with trial-based economic evaluations possibly biasing internal validity of the review. Conference abstracts, protocol papers, case studies, non-peer-reviewed articles and articles in languages other than English, German or Dutch, pilot studies, feasibility studies, cohort, observational and cross-sectional studies were excluded.

### Study selection and extraction

The first author (C.B.) completed the literature search. Two independent reviewers (C.B. and F.K.) screened abstracts for inclusion of the publications in the review and when the abstract did not provide sufficient information to determine eligibility the full text was read. Any disagreement between C.B. and F.K. was resolved by discussion, while a third reviewer (D.D.E.) was consulted if C.B. and F.K. could not reach consensus. Included studies were classified as cost-effectiveness or cost-utility studies for the prevention or treatment of identified SUD conditions. A narrative review of the characteristics of included studies was done. Information to be extracted included SUD condition; study sample; intervention; comparator (e.g. treatment as usual, face-to-face treatment); outcome measurement and incremental cost-effectiveness ratio (ICER) results; the type of cost-effectiveness study performed (including cost-effectiveness analysis [CEA, where outcomes are expressed in clinical units, such as clinical scales] and cost-utility analysis [CUA, where outcomes are presented in generic units such as quality-adjusted life years (QALYs)]; study perspective (e.g. societal, health care, provider) and time horizon. To compare costs or ICERs across all studies, all costs were converted into Euro. First, by using country-specific gross domestic product inflators the currency of the study was converted into 2014 equivalent (e.g. the average year of included studies). Second, purchasing power parities (PPPs) were used to convert to Euro (e.g. Euro area, 19 countries) for the studies, which reported the costs in non-Euro currencies.^21^ According to the National Institute for Health and Clinical Excellence [NICE] a willingness-to-pay (WTP) ceiling of £20 000–£30 000 should be applied for gaining one QALY. This WTP ceiling range corresponds with €24 600–€36 900 (PPP converted and indexed for the reference year 2014) and were used in our review to aid interpretation of the results.

### Quality assessment

The quality of included studies were assessed by the 24-item Checklist of the Consolidated Health Economic Evaluation Reporting Standards (CHEERS).^22^ Quality assessment were completed for each included study by C.B. and reviewed by F.K. In case of disagreement a third reviewer (D.D.E.) was consulted. A scoring system to classify study quality was used where a point was given for each criterion met. A point was withheld where criteria were not fulfilled completely. A score of quality assessment was given based on the percentage of criteria met by each study that ranged from 0% to 100%. To report on quality of reporting, four categories were used: excellent (100%), good (75—99%), average (50—74%) and poor (< 50%) quality of reporting. In addition, we assessed the quality of included studies using the Cochrane Collaboration’s tool for assessing risk of bias. Results can be found in the Supplementary Data.

## Results

### Study selection

**Figure 1:**
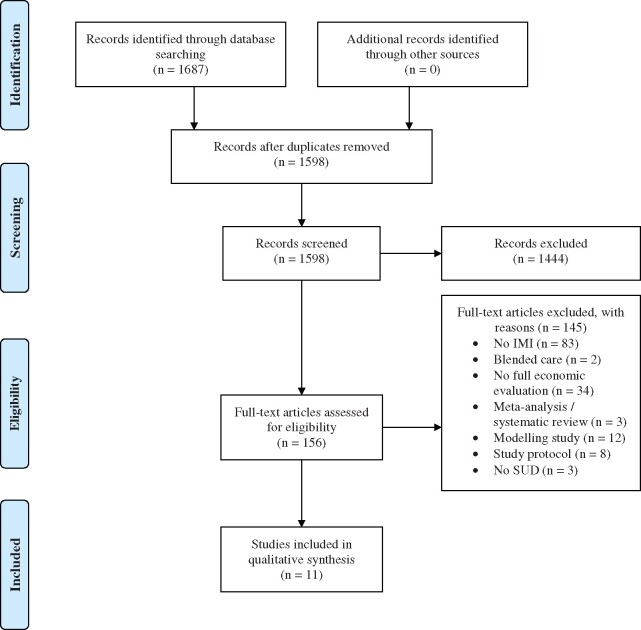
PRISMA flow diagram

The literature search identified 1687 articles. After removal of duplicates, a total of 1598 abstracts were screened and 156 full text articles were retrieved for further consideration. Of those, 11 studies met inclusion criteria including 14 comparisons (see figure 1). In the excluded studies, for which full text were retrieved, 83 studies did not evaluate an IMI (i.e. booklets/letters were sent by post), in 2 studies the IMI was offered as blended care, 34 studies did not meet criteria for a full economic evaluation (i.e. costs were examined but not in relation to the clinical outcome), 3 studies were meta-analyses or systematic reviews, 12 studies evaluated economic effects with a decision-analytical model, 8 studies reported on the design of an economic evaluation and 3 studies did not evaluate an IMI for SUDs.

### Characteristics of included studies

A description of included studies is provided in table 1. Four of the 11 studies were conducted in The Netherlands, 4 in the USA, 1 study in Denmark, 1 in the United Kingdom and another in Italy. All studies targeted adults, except one, which was directed at adolescents.^23^ A total of 18 652 participants were included across the included studies. Target conditions were alcohol use disorder (four studies) and tobacco smoking (five studies) whereas two studies included any SUD. Types of economic evaluations included cost-effectiveness (nine studies) and cost-utility analyses (seven studies), which were performed from a societal (four studies), provider (four studies), health care (four studies) or payer’s (two studies) perspective. Most of the interventions were based on theories of motivational and behaviour change or cognitive behavioural therapy. Comparators included treatment as usual (TAU) (four studies), (online) psycho-education (four studies), face-to-face treatment (two studies) and an IMI with a different guidance format compared with the IMI under study (one study). We refer to TAU when a comparator condition was either explicitly mentioned as TAU in the published papers or represented standard care/current best practice according to available treatment guidelines.

**Table 1:** Main characteristics of included studies

Author	Country	Target condition	Sample	Sample size	Type of study	Study perspective	Time horizon
Blankers et al.^24^	Netherlands	Alcohol use disorder	Adults	136	CEA	Societal	6 months
					CUA	Health care	
Drost et al.^23^	Netherlands	Alcohol use disorder	Adolescents	2493	CEA	Societal	4 months
						Health care	
Hunter et al.^25^	Italy	Alcohol use disorder	Adults	763	CUA	Italian NHS	12 months
						NHS	
Wallace et al.^26^	UK	Alcohol use disorder	Adults	7935	CUA	Not clear	Not clear
Calhoun et al.^27^	USA	Smoking	Veterans	413	CEA	Payer	12 months
					CUA		
Graham et al.^28^	USA	Smoking	Adults	2005	CEA	Payer	18 months
Skov-Ettrup et al.^29^	Denmark	Smoking	Adults	1810	CEA	Not clear	12 months
Smit et al.^30^	Netherlands	Smoking	Adults	414	CEA	Societal	12 months
					CUA		
Stanczyk et al.^31^	Netherlands	Smoking	Adults	2099	CEA	Societal	12 months
					CUA		
Murphy et al.^32^	USA	Any SUD	Adults	507	CEA	Provider	12 weeks
					CUA		
Olmstead et al.^33^	USA	Any SUD	Adults	77	CEA	Provider	8 weeks

Notes: CEA, cost-effectiveness analysis; CUA, cost-utility analysis; NHS, National Health Service; SUD, substance use disorder.

### Findings of included studies

Characteristics of and results for economic evaluations of IMIs for SUDs can be found in table 2. Table 2 represents the actual costs that were published in the specific papers whereas costs presented throughout the paper are stated in standardized metrics (Euros) to compare costs across the published studies.

**Table 2:** Characteristics of and results for economic evaluations of internet-based interventions for substance use disorder

Author	Treatment alternatives	Effect measurement and valuation	ICER results^a^	Conclusion
Alcohol				
Blankers et al.^24^	Guided CBT + motivational interviewing Unguided CBT + motivational interviewing	Treatment response (AUDIT); QALYs (EQ-5D)	Societal perspective: CEA: median ICER = €3683/treatment responder CUA: median ICER = €14 710/QALY gained At a WTP of €20 000/QALY, the probability that guided intervention was more cost effective than the unguided intervention was 60% Dutch health care perspective: CEA: median ICER = €1157/treatment responder CUA: median ICER = €4693/QALY gained	Guided CBT + motivational interviewing offers better value for money than unguided self-help and might be considered as a treatment option
Drost et al.^23^	Unguided web-based intervention with computer-tailored feedback TAU	Mean number of glasses of alcohol per week Number of binge drinking occasions	Societal perspective: Mean ICER = €62/incremental reduction of one glass of alcohol per week Mean ICER = €144/binge drinking occasion per 30 days Dutch health care perspective: Mean ICER = €40/incremental reduction of one glass of alcohol per week Mean ICER = €79/binge drinking occasion per 30 days	An unguided intervention with computer-tailored feedback could be a cost-effective strategy to target alcohol use disorder and binge drinking among adolescents
Hunter et al.^25^	Interactive alcohol reduction website TAU (brief f2f intervention provided by GP)	QALYs (EQ-5D)	No ICER reported At a WTP of €250 000/QALY gained, the probability that the interactive website was cost-effective (excluding website developing costs) was 84% Applying British NHS costs (only including intervention costs), the probability that the website was cost-effective at a WTP of £25 000/QALY gained was 75%	Facilitated access to an interactive website to reduce alcohol use disorder costs less than TAU with no worse outcomes. The lower cost of facilitated access may facilitate the increase in provision of interventions for alcohol use disorder
Wallace et al.^26^	Interactive alcohol reduction website Online psycho-education	QALYs (EQ-5D)	CEA: no difference in alcohol consumption CUA: no difference in incremental QALYs Incremental costs of £26.17 in favour of online psycho-education	Findings did not provide support for the hypothesis that access to an interactive website offers additional benefit over online psycho-education
Smoking				
Calhoun et al.^27^	All participants: nicotine replacement therapy Unguided online tobacco cessation programme Specialty clinic-based smoking cessation	12-month quit rates; QALYs (not clear)	ICER not reported	There were no statistical differences in overall quit rates or cost-effectiveness between veterans referred to clinic-based specialty care or to an unguided online tobacco cessation programme
Graham et al.^28^	Unguided online tobacco cessation programme Unguided online tobacco cessation programme + proactive telephone counselling Online psycho-education	Number of quitters (abstinence defined as 30-day single-point prevalence)	Unguided online tobacco cessation programme dominated by online psycho-education Unguided online tobacco cessation programme + phone counselling compared with online cessation programme alone: mean ICER = $3781/additional quitter	Online psycho-education had the lowest cost per quitter at all time points. The most cost-effective intervention was the unguided online tobacco cessation programme combined with proactive telephone counselling
Skov-Ettrup et al.^29^	Unguided internet- and text-based smoking cessation programme Proactive telephone counselling Reactive telephone counselling Self-help booklet (control)	Prolonged abstinence measured at 12-month follow-up	Unguided intervention vs. self-help booklet: mean ICER = £20/additional quitter	No clear evidence of an effect of the unguided internet- and text-based smoking cessation programme was found compared with the self-help booklet
Smit et al.^30^	Unguided internet-based multiple computer-tailored intervention + f2f counselling by practice nurse TAU	Prolonged abstinence measured at 12-month follow-up; QALYs (EQ-5D)	Societal perspectiveCEA: Unguided intervention dominates intervention + f2f counselling by practice nurse Unguided intervention vs. TAU: mean ICER = €5100/additional quitter CUA: TAU dominates unguided intervention Unguided intervention + f2f counselling vs. TAU: mean ICER = €40 300/QALY gained	Cost-effectiveness analyses revealed that the unguided intervention would probably be the most cost-effective treatment of the three treatments under study Cost-utility results suggested that TAU would probably be the most preferable of the three treatments
Stanczyk et al.^31^	Video-based multiple computer-tailored intervention Text-based multiple computer-tailored intervention Online psycho-education (control)	Prolonged abstinence measured at 12-month follow-up QALYs (EQ-5D)	Societal perspective CEA: Video-based vs. online psycho-education: mean ICER = €1500/abstinent responder Text-based vs. online psycho-education: mean ICER = €50 400/abstinent responder Video-based dominates text-based intervention CUA: Video-based vs. online psycho-education: mean ICER = €60 000/QALY gained Text-based intervention is dominated by online psycho-education and video-based intervention	The video-based intervention was the most cost-effective treatment for smoking abstinence after 12 months. Cost-utility analyses showed that online psycho-education seemed to be the most preferable treatment option
Any SUD				
Murphy et al.^32^	Therapeutic Education System (internet-based community reinforcement approach + computer-assisted contingency management) + TAU TAU	Abstinent year QALY (EQ-5D)	Provider’s perspective CEA: Mean ICER = $9073/abstinent-year CUA: TAU+TES is dominated by TAU	The probability that TES+TAU is considered to be cost-effective from the provider’s perspective at a WTP of $20 000/abstinent year is 95%
Olmstead et al.^33^ (2010)	cCBT+TAU TAU	Drug-free specimen	Provider’s perspectiveMean ICER = $21/drug-free specimen At a WTP of $0, the probability that cCBT+TAU is considered cost-effective was 14% At a WTP of $75, this probability was 90%	cCBT+TAU appears to be a good value from the provider’s perspective

Notes: CBT, cognitive behavioural therapy; QALYs, quality-adjusted life years; CEA, cost-effectiveness analysis; CUA, cost-utility analysis; ICER, incremental cost-effectiveness ratio; TAU, treatment-as-usual; f2f, face-to-face; GP, General Practitioner; NHS, National Health Service; cCBT, computer-based cognitive behavioural therapy.

a Table 2 represents the actual costs that are published in the specific papers.

### Alcohol use disorder

Two of the four studies in IMIs for alcohol use disorder conducted a CEA, from both the societal and the Dutch health care perspective.^23^^,^^24^ Three of the four studies performed a CUA,^24–26^ with one study applying both a societal and health care perspective,^24^ one study deploying a health care perspective^25^ and one study not clearly stating the study perspective.^26^

#### Cost-effectiveness analysis

- Blankers et al.^24^ took the Dutch health care perspective as well as the societal perspective to evaluate the economic benefit of a guided IMI compared with an unguided IMI in adults concluding that the guided IMI based on CBT and motivational interviewing techniques offered good value for money compared with the unguided IMI within a 6-month time horizon. From the societal perspective (Dutch health care perspective), the reported median ICER was €3817 (€1254) per additional treatment responder concluding that above a WTP of approximately €4000 (€1300) per additional responder, the guided IMI is considered cost-effective compared with the unguided IMI. For the ICERs expressed in local currency units (see table 2).
- Drost et al.^23^ showed that an unguided IMI with computer-tailored feedback based on theories of motivational and behaviour change could be a cost-effective way of targeting problematic alcohol use and binge drinking among adolescents. From the societal perspective (Dutch health care perspective), the ICER/reduction of one glass of alcohol per week was €62 (€40) and €144 (€79) for one binge drinking occasion per 30 days. With increasing WTPs (up to €500), the probability of the IMI being cost-effective increased to approximately 80% for both outcomes from both perspectives.

#### Cost-utility analysis

Two studies evaluated the cost-effectiveness of an unguided interactive IMI based on theories of motivational and behaviour change and CBT.^25^^,^^26^

- From the societal perspective, Blankers et al.^24^ found that the guided IMI resulted in better health effects. An additional QALY was gained at a median incremental cost of €15 948. At a WTP of €21 683/QALY gained, the probability that the guided IMI was considered to be more cost-effective than the unguided IMI was 60%. From the Dutch health care perspective, the median ICER was €5088/QALY gained.

- Hunter et al.^25^ in their Italian study observed that referral of patients to an unguided interactive IMI compared with referral to a brief face-to-face intervention delivered by the General Practitioner (GP; TAU) was associated with additional benefits at 12-month follow-up. From the perspective of the Italian NHS, the IMI had a probability of 84% of being cost-effective at a WTP of €25 000/QALY gained. Applying English NHS costs, this probability was 75% at a WTP of €31 000/QALY gained.
- In contrast, Wallace et al.^26^ could not find support for the hypothesis that the unguided interactive IMI offered additional benefit over online psycho-education in terms of clinical effectiveness, with incremental costs being slightly in favour of online psycho-education. However, in this study neither the study perspective nor the time horizon was clear.

In general, findings of the two cost-effectiveness analyses suggested that IMIs for alcohol use disorder provide good value for money. Cost-utility analyses supported these findings. Reported ICERs per QALY gained were all below (or within) the WTP range of €24 600–€36 900. Probabilities that IMIs were cost-effective ranged from 60% to 84% with higher probabilities when a public health care perspective was taken compared with the societal perspective.

### Tobacco smoking

All five studies in IMIs for smoking cessation employed a CEA with incremental costs per quitter with at least a 12-month time horizon,^27–31^ with one study having a prolonged follow-up of 18 months.^28^ Two studies performed the CEA from the payer’s perspective,^27^^,^^28^ two studies from the societal perspective,^30^^,^^31^ and in one study the perspective was not clear.^29^ Three studies additionally evaluated incremental costs per QALY gained, from either the payer’s perspective^27^ or the societal perspective.^30^^,^^31^

#### Cost-effectiveness analysis

Two studies investigated the cost-effectiveness of an unguided IMI from the payer’s perspective:

- Skov-Ettrup et al.^29^ showed an ICER of €25/additional quitter when comparing an unguided IMI to a self-help booklet. However, the study perspective was unclear and incremental differences in effectiveness were not statistically significant.

- Calhoun et al.^27^ did not find statistically significant differences in incremental abstinence rates between the IMI paired with a tele-medicine clinic for nicotine replacement therapy and an assisted referral to speciality smoking cessation clinic-based care, thus no ICER was calculated.
- Graham et al.^28^ found that the unguided IMI was dominated by online psycho-education. Enhancing the IMI with telephone counselling resulted in higher costs but also greater effects, thus the enhanced IMI was considered a cost-effective approach to smoking cessation compared with the IMI alone with an ICER of €2897/additional quitter.

Two studies evaluated various versions of an unguided IMI with computer-tailored feedback from the societal perspective.^30^^,^^31^

- In the first study by Smit et al.,^30^ an unguided IMI coupled with computer-tailored feedback or face-to-face counselling by a practice nurse, respectively, was compared with TAU. The study revealed that the IMI with computer-tailored feedback would probably be the most cost-effective treatment option when incremental costs per additional quitter were evaluated (i.e. 78% at a WTP of €19 038). In contrast to Graham et al.,^28^ Smit et al.^30^ found that the IMI dominated the combination of IMI and face-to-face counselling meaning that the IMI generated larger health effects at lower costs compared with the employment of a nurse to provide feedback. Compared with TAU, the ICER for the IMI was €5394/additional quitter.^30^ However, although Smit et al.^30^ stated that they employed a societal perspective; they did not include indirect costs.
- The second study by Stanczyk et al.^31^ found that at a WTP of €18 058/quitter, an unguided IMI with video-based computer-tailored feedback was the most cost-effective treatment option for smoking cessation (i.e. 70%) compared with text-based computer-tailored feedback (i.e. 11%) and online psycho-education (i.e. 20%). The ICER for the IMI with video-based feedback compared with online psycho-education was €1505/additional quitter and €50 561/additional quitter for the IMI with text-based feedback compared with online psycho-education.

#### Cost-utility analysis

In general, results from cost-effectiveness studies conducted from the payer’s perspective did not suggest an economic merit of IMIs. Findings from cost-effectiveness analyses employing a societal perspective suggested that IMIs for smoking cessation have an acceptable likelihood of being cost-effective compared with either TAU or online psycho-education with probabilities ranging from 70% to 78%. Cost-utility analyses, however, did not support the hypothesis that IMIs are cost-effective as reported ICERs were well above the acceptable cost-effectiveness threshold of €24 600–€36 900.

- As in the CEA, Calhoun et al.^27^ did not find statistically significant differences in incremental effectiveness (i.e. QALY gained) between the IMI paired with a tele-medicine clinic for nicotine replacement therapy and an assisted referral to speciality smoking cessation clinic-based care from the payer’s perspective, thus no ICER was calculated.
- Smit et al.^30^ showed in cost-utility analyses from the societal perspective that TAU would probably be the most preferable treatment option with a probability of 64% at a WTP of €19 038/QALY gained. The IMI alone was dominated by TAU and the ICER for the nurse-led feedback compared with TAU was €42 625/QALY gained. Thus, at a WTP of approximately €43 000, TAU and the IMI coupled with counselling would be equally preferable.
- Stanczyk et al.^31^ revealed in cost-utility analyses that from the societal perspective at a WTP of €18 058, online psycho-education seemed to be the most preferable treatment choice (i.e. 43%), followed by the IMI with video-based feedback (i.e. 39%) and the IMI with text-based feedback (i.e. 18%). Compared with online psycho-education, the ICER for the IMI with video-based feedback was €60 192/QALY gained whereas the IMI with text-based feedback was dominated by both the IMI with video-based feedback and online psycho-education.

### Any SUD

Two studies evaluated IMIs from a provider’s perspective with a post-treatment time horizon that were targeted to patients suffering from any SUD (i.e. stimulants, opioids, alcohol and marijuana).

- Murphy et al.^32^ found an ICER of a Therapeutic Education System (TES) including an internet-based reinforcement approach and computer-assisted contingency management as an adjunct to TAU compared with TAU alone of €6745/abstinent year. At a WTP of €14 869, the probability of TES plus TAU to be considered cost-effective was 95%. Cost-utility analyses revealed that TAU dominated TES plus TAU.
- Olmstead et al.^33^ showed that an unguided IMI based on CBT plus TAU appeared to be good value for money compared with TAU alone. The ICER was €1340/drug-free specimen, with a probability that the IMI plus TAU was more cost-effective than TAU of 14% at a WTP of €0 and 90% at a WTP of €4786.

Cost-effectiveness analyses on IMIs targeting any SUD showed that IMIs could provide good value for money from a provider’s perspective within a short time horizon when a provider is willing to pay €5000–€15 000 for a drug-free specimen or abstinent year, respectively. As for alcohol use disorder and tobacco smoking, findings of the CUA did not support the hypothesis that IMIs are a more cost-effective than TAU.

### Quality assessment

All of the studies included in this review met over 50% of quality criteria indicating that the quality of reporting was at least of average quality (see table 3). The mean percentage of items met in the studies was 82%. One study was classified as ‘excellent’.^25^ Seven studies fulfilled criteria of good quality of reporting.^23^^,^^24^^,^^28^^,^^30–33^ Common reasons why these studies did not achieve 100% of the criteria were a lack of reporting the choice of discount rate, information on valuation of preference-based outcomes and reporting of parameters required to calculate overall costs and consequences and their associated values. Three studies were only classified as average quality of reporting with the lowest value of 62% of met criteria.^26^^,^^27^^,^^29^ Common criteria that were not met included the description of analytical methods and characterization of uncertainty.

**Table 3:** Quality assessment with CHEERS checklist

CHEERS statement checklist	Blankers et al.^24^	Calhoun et al.^27^	Drost et al.^23^	Graham et al.^28^	Hunter et al.^25^	Murphy et al.^32^	Olmstead et al.^33^	Skov-Ettrup et al.^29^	Smit et al.^30^	Stanczyk et al.^31^	Wallace et al.^26^
Title and abstract											
Title	1	0	1	1	1	1	1	0	1	1	0
Abstract	1	0	1	1	1	1	1	0	1	1	0
Introduction											
Background and objectives	1	1	1	1	1	1	1	1	1	1	1
Methods											
Target population and subgroups	1	1	1	1	1	1	1	1	1	1	1
Setting and location	1	1	1	1	1	1	1	1	1	1	0
Study perspective	1	1	1	1	1	1	1	1	1	1	0
Comparators	1	1	1	1	1	1	1	1	1	1	1
Time horizon	1	1	1	1	1	1	1	1	1	1	0
Discount rate	0	0	0	1	1	0	0	0	1	1	0
Choice of health outcomes	1	1	1	1	1	1	1	1	1	1	1
Measurement of effectiveness	1	1	1	1	1	1	1	1	1	1	1
Measurement and valuation of preference based outcomes	1	0	N/A	0	1	0	N/A	N/A	0	1	1
Estimating resources and costs	1	1	1	1	1	1	1	1	1	1	1
Currency, price data and conversion	1	0	1	0	1	1	0	1	1	1	1
Choice of model	N/A	N/A	N/A	N/A	N/A	N/A	N/A	N/A	N/A	N/A	N/A
Assumptions	N/A	N/A	N/A	N/A	N/A	N/A	N/A	N/A	N/A	N/A	N/A
Analytical methods	1	0	1	0	1	1	1	0	1	1	0
Results								0			
Study parameters	1	0	1	1	1	1	0	0	0	0	1
Incremental costs and outcomes	1	1	1	1	1	1	1	1	1	1	1
Characterizing uncertainty	1	0	1	0	1	1	1	0	1	1	0
Characterizing heterogeneity	N/A	N/A	1	1	N/A	N/A	N/A	0	N/A	N/A	0
Discussion											
Study findings, limitations, generalizability and current knowledge	1	1	1	1	1	1	1	1	1	1	1
Other											
Source of funding	1	1	1	1	1	0	0	1	1	1	1
Conflicts of interest	1	1	1	1	1	1	0	1	1	1	1
CHEERS score	20/21	13/21	20/21	18/22	21/21	18/21	16/21	13/21	19/21	19/21	14/22
Quality of reporting	95% good	62% average	95% good	82% good	100% excellent	86% good	76% good	62% average	90% good	90% good	64% average

Notes: CHEERS, Checklist of the Consolidated Health Economic Evaluation Reporting Standards; N/A, the item is not applicable.

## Discussion

### Summary of main findings

The aim of this review was to provide an overview of trial-based health-economic evaluation studies of IMIs for SUD compared with control conditions. Findings of cost-effectiveness analyses suggested that IMIs for SUDs compared with TAU provide good value for money. One of the two studies comparing an IMI to a face-to-face treatment found an acceptable likelihood of the IMI to be cost-effective^25^ whereas the other study did not find significant differences in the clinical outcome.^27^ Compared with (online) psycho-education, evidence for an additional benefit of IMIs is less clear, as only one of four studies showed that an IMI had a higher probability of being more cost-effective than psycho-education. Regarding cost-utility (i.e. incremental costs per QALY gained), except for one study,^25^ results suggested that TAU and online psycho-education would probably be more preferable than IMIs. One study provided supporting evidence that a guided IMI offers better value for money than unguided self-help, both in cost-effectiveness and cost-utility analyses.^24^ Due to the heterogeneity in types of IMIs, sorts of ‘treatment-as-usual’ and targeted SUD populations, no single and general conclusion about the cost-effectiveness of IMIs can be presented in this fragmented research field.

### Quality of included studies

Some methodological limitations need to be mentioned. Many studies provided insufficient information on parameters required to calculate overall costs and consequences and their associated values. Of the 11 studies included in the review, six did not report on these parameters^27^^,^^29–31^^,^^33^ thereby hindering transparent interpretation of their evaluation. Furthermore, the analytical methods including handling sampling uncertainty were not clearly reported and justified in four studies.^26–29^ Presenting cost-effectiveness planes and cost-effectiveness acceptability curves may be appropriate ways to present stochastic uncertainty due to sample error, but those graphs were not always produced in the reviewed studies. In addition, one of the studies that adopted a societal perspective did not include productivity costs in the analyses.^30^

### Comparison with prior research

The current review found that the number of economic evaluations alongside randomized controlled trials of IMIs for SUDs has significantly increased since 2014. Findings from our systematic review support findings from previous reviews that IMIs could be a cost-effective way to target SUDs and increase the reach of effective treatments.^16^^,^^18^^,^^34^^,^^35^

### Strength and limitations

One strength of this review was the comprehensive database search. Another important strength was following recommended steps for converting ICERs to the same currency for the same year to enable comparisons between studies. Yet, expressing ICERs in the same currency still does not account for different collection and valuation of costs between studies. Some limitations are to be mentioned. Included studies used a variability of methods, such as varying time horizons, comparators and study perspectives, hindering comparison of results. For example, next to differences in costs due to different study perspectives, studies differed with regard to the inclusion and exclusion of IMI development costs. Whereas some studies included these costs, in other studies these costs were considered as sunk costs. In addition, only four studies included TAU as alternative, which restricts the interpretation as to whether IMIs are cost-effective compared with standard care. Another limitation is that conclusions about long-term cost-effectiveness of IMIs cannot be made since only 1 out of 11 studies included a time frame beyond 18 months.

### Practical implications and future research

The economic evaluations incorporated in this review comparing an IMI to TAU mostly demonstrated favourable cost-effectiveness across varying target populations. IMIs cost less than face-to-face health care services for SUDs (i.e. counselling by a GP or practice nurse) and showed no worse clinical outcomes (i.e. abstinence rates). The lower cost of IMIs, particularly with regard to investment of therapists’ time, may facilitate the increase in provision of interventions for alcohol use disorder and smoking cessation. However, studies lacked evidence for cost-effectiveness of IMIs when QALYs were included as the outcome of interest. A possible explanation could be that a time horizon of maximal 12 months was not sufficiently long for the beneficial effects of IMIs on abstinence to be translated into detectable changes in quality of life. More specifically, it needs to be taken into account that owing to the toxicity of tobacco and alcohol their adverse health effects linger on in former smokers and former drinkers for many years. Thus, cessation/abstinence does not translate itself immediately into health gains. For that, randomized controlled trials including both sustained/prolonged abstinence plus longer follow-up times are needed. However, this might not be feasible in any case. Modelling studies could extrapolate trial findings and thus give an indication about the long-term cost-effectiveness of IMIs for SUDs. However, to the best of our knowledge, no systematic review on model-based economic evaluations of IMIs for SUDs has been conducted so far. In addition, evidence for the cost-effectiveness of IMIs for SUDs other than alcohol and tobacco use is scarce. There is accumulating evidence for IMIs for cannabis use.^11^ However, the cost-effectiveness of such IMIs has not yet been evaluated.

## Conclusion

In conclusion, in the small sample of studies with both outcome data and economic evaluations, IMIs for SUDs showed a high probability of being more cost-effective than treatment as usual (e.g. lower costs per additional abstinent person). However, incremental cost-utility analyses based on QALYs gained were less convincing, perhaps owing to the persistence of adverse health effects of alcohol and tobacco after (prolonged) cessation. With an increasing pressure on budgets of health care systems, strategies to disseminate effective interventions at affordable costs are direly needed. Results of this review are promising and support the notion that employment of IMIs might be a cost-effective strategy to scale-up existing evidence-based treatments for SUDs.

## Supplementary data

Supplementary data are available at *EURPUB* online.

## Funding

This project has received funding from Horizon 2020 (EU Research and Innovation programme) under grant agreement No 634757.

*Conflicts of interest*: None declared.

**Key points**

- Due to the heterogeneity in types of internet- and mobile-based interventions, sorts of ‘treatment-as-usual’ and targeted conditions, no single and general conclusion about the cost-effectiveness of internet interventions can be presented in this fragmented research field.
- The economic evaluations included in this systematic review comparing an internet- and mobile-based intervention to a control condition mostly demonstrated favourable cost-effectiveness across varying target conditions.
- Results of this review are promising and support the notion that employment of internet- and mobile-based interventions might have the potential as cost-effective strategy to scale-up existing evidence-based treatments for substance use disorders.

## Supplementary Material

ckz022_Supplementary_Data
