# Supplementary material for: A systematic review of trial-based economic evaluations of internet- and mobile-based interventions for substance use disorders
Source: Eur J Public Health. 2019 Jul 12;31(Suppl 1):i19–28. doi: 10.1093/eurpub/ckz022 (PMC8266535; doi:10.1093/eurpub/ckz022)
Supplement: ckz022_Supplementary_Data [file ckz022_supplementary_data.zip › ckz022-suppl_data/Supplement 1_eTable_Risk of bias assessment.doc]

This supplemental material has been provided by the authors to give readers additional information about their work.

**eTable 1.** Cochrane Collaboration’s tool for assessing risk of bias

|  | Blankers (24) (2012) | Calhoun (27) (2016) | Drost (23) (2016) | Graham (28) (2013) | Hunter (25) (2017) | Murphy (32) (2016) | Olmstead (33) (2010) | Skov-Ettrup (29) (2016) | Smit  (30) (2013) | Stanczyk (31) (2014) | Wallace (26) (2011) |
| --- | --- | --- | --- | --- | --- | --- | --- | --- | --- | --- | --- |
| Random sequence generation | Low risk | Unclear risk | Low risk | Low risk | Unclear risk | Low risk | Low risk | High risk | Low risk | Unclear risk | Low risk |
| Allocation concealment | Low risk | Unclear risk | Unclear risk | Unclear risk | Low risk | Low risk | Unclear risk | Low risk | Low risk | Low risk | Low risk |
| Blinding of participants and personnel | Low risk | Unclear risk | Unclear risk | Low risk | Low risk | Low risk | Low risk | Low risk | Low risk | Low risk | Low risk |
| Blinding of outcome assessment | Low risk | Unclear risk | Low risk | High risk | Low risk | Low risk | Low risk | Low risk | Low risk | Low risk | Low risk |
| Incomplete outcome data | Low risk | Low risk | Unclear risk | Low risk | Unclear risk | Low risk | Low risk | Low risk | Low risk | Low risk | Low risk |
| Selective reporting | Low risk | Unclear risk | Unclear risk | Unclear risk | Low risk | Low risk | Low risk | Low risk | Low risk | Low risk | Low risk |
| Other bias | Low risk | High risk No trial registration reported | Low risk | High risk Change in primary outcome compared to trial registration | Low risk | Low risk | Low risk | Low risk | Low risk | Low risk | Low risk |
| **Summary** | **Low risk** of bias for all domains | **Low risk** of bias for 1 domain;  **Unclear** **risk** of bias for 5 domains; **High risk** of bias for 1 domain | **Low risk** of bias for 3 domains;  **Unclear** risk of bias for 4 domains | **Low risk** of bias for 3 domain;  **Unclear risk** of bias for 2 domains; **High risk** of bias for 2 domain | **Low risk** of bias for 5 domains;  **Unclear risk** of bias for 2 domains | **Low risk** of bias for all domains | **Low risk** of bias for 6 domains;  **Unclear risk** of bias for 1 domain | **Low risk** of bias for 6 domains;  **High risk** of bias for 1 domain | **Low risk** of bias for all domains | **Low risk** of bias for 6 domains;  **Unclear risk** of bias for 1 domain | **Low risk** of bias for all domains |

Most information is from studies at low or unclear risk of bias. Four of the eleven studies showed low risk of bias for all key domains (2 studies on IMIs for alcohol use disorder, 1 study on IMI for smoking cessation, 1 study on IMI for any SUD). Low risk of bias is unlikely to seriously alter the results. Six of the included studies showed an unclear risk of bias for one or more key domains (3 studies on IMI for smoking cessation, 2 studies on IMIs for alcohol use disorder, 1 study on any SUD). Unclear risk of bias raises some doubt about the results found in the studies concerned. In three studies, high risk of bias was detected in one or more key domains that could seriously weaken the confidence in the results. All three studies evaluated IMIs for smoking cessation. However, the proportion of information from studies at high risk of bias is considered not sufficient to affect the interpretation of results.
