# Supplementary material for: A systematic review of trial-based economic evaluations of internet- and mobile-based interventions for substance use disorders
Source: Eur J Public Health. 2019 Jul 12;31(Suppl 1):i19–28. doi: 10.1093/eurpub/ckz022 (PMC8266535; doi:10.1093/eurpub/ckz022)
Supplement: ckz022_Supplementary_Data [file ckz022_supplementary_data.zip › ckz022-suppl_data/Supplement 2_Search String.doc]

**Supplement 2**

This supplemental material has been provided by the authors to give readers additional information about their work.

**PubMed search string**

(cost-effective[TIAB] OR cost-effectiveness OR cost-utility[TIAB] OR economic evaluation[TIAB] OR cost-[TIAB]) AND (addiction[TI] OR drug[TI] OR substance[TI] OR alcohol[TI] OR tobacco[TI] OR smoking[TI] OR cannabis[TI] OR marijuana[TI] OR heroin[TI] OR cocaine[TI] OR opiate[TI] OR non-opiate[TI] OR use[TI] OR dependence[TI] OR abuse[TI] OR misuse[TI] OR problem[TI] OR cessation[TI]) AND (telemedicine[TI] OR digital[TI] OR text-message[TI] OR mobile[TI] OR smartphone[TI] OR computer-assisted[TI] OR computer-tailored[TI] OR internet[TI] OR online[TI] OR online-based[TI] OR Web[TI] OR web-based[TI] OR internet-based[TI] OR computer*[TI] OR therap*[TI] OR treatment[TI] OR prevention[TI] OR intervention*[TI] OR self-help[TI] OR e-therap*[TI] OR etherap*[TI] OR behavio*[TIAB] OR brief intervention[TIAB] OR motivational interviewing[TIAB]) AND ((controlled clinical trial)[TIAB] OR random* OR (random* controlled trial) OR RCT) NOT (study protocol[TI] OR meta-analysis[TI] OR systematic review[TI])
